# Reasons and pathways of first-time consultations at child and adolescent mental health services in Italy: an observational study

**DOI:** 10.1186/s13034-015-0060-9

**Published:** 2015-08-19

**Authors:** Laura Pedrini, Davide Sisti, Alessandra Tiberti, Antonio Preti, Michela Fabiani, Linda Ferraresi, Stefano Palazzi, Roberto Parisi, Cosimo Ricciutello, Marco B. L. Rocchi, Antonella Squarcia, Stefano Trebbi, Andrea Tullini, Giovanni De Girolamo

**Affiliations:** IRCCS San Giovanni di Dio Fatebenefratelli di Brescia, via Pilastroni 4, Brescia, 25125 Italy; Dipartimento di Scienze Biomolecolari, Università degli Studi di Urbino “Carlo Bo”, Piazza Rinascimento 6, Urbino (PU), 61029 Italy; Servizio Neuropsichiatria Infanzia e Adolescenza, Spedali Civili di Brescia, P.le Spedali Civili 1, Brescia, 25123 Italy; Centro di Psichiatria di Consulenza e Psicosomatica dell’ Azienda Ospedaliero Universitaria di Cagliari, via Ospedale, 09124 Cagliari, Italy; Dipartimento di Pedagogia Psicologia e Filosofia, Università di Cagliari, Via Mirrionis 1, Cagliari, 09123 Italy; UONPIA, AUSL di Reggio Emilia, Via Amendola 2, Reggio Emilia, 42100 Italy; UONPIA, AUSL di Modena, Via Alessandrini 2, Sassuolo, 41049 Italy; UONPIA, AUSL di Ferrara, via Messidoro 20, Ferrara, 44124 Italy; UONPIA, AUSL di Piacenza, Corso Vittorio Emanuele 169, Piacenza, 29100 Italy; UONPIA, AUSL di Imola, Viale Amendola 8, Imola (BO), 40026 Italy; Servizio N.P.E.E., Az. USL di Parma, Via Mazzini 2, Parma, 43100 Italy; NPI, Az. USL di Bologna, Via Crisalidi 2, Vado (Monzuno), BO 40036 Italy; Servizio N.P.E.E., Az. USL di Rimini, Via Coriano 38, Rimini, 47921 Italy

**Keywords:** Help-seeking, Mental health, Child, Adolescent, Health services

## Abstract

**Background:**

An increasing number of young people have made contact with the Child and Adolescent Mental Health Services (CAMHS). However, only a small proportion of the population with emotional problems, actually seek specialized care. Research concerning the help-seeking process and pathways to care of a clinical sample could help to develop effective health policies to facilitate access to specialized care.

**Aim:**

To analyze the access pattern for CAMHS, reasons of contact and care pathways of a consecutive sample of first-time patients. Our aim was to analyze the association between source of referral, socio-demographic and clinical variables.

**Methods:**

Standardized assessment instruments and information concerning access patterns and care pathways were collected from 399 patients at first-time contact with CAMHS in a Northern Italian Region.

**Results:**

Most patients were referred to CAMHS by school teachers (36 %) or health professionals (32 %), while only 17 % of the parents sought help by themselves. School issues (50 %) and emotional problems (17 %) were the most frequent reasons for contact. The proportion of first-time contacts with no diagnosis of mental disorder at their first consultation did not differ by source of referral. Parents of children who did not receive a clinical diagnosis of mental disorders described them as “psychosocially impaired” and their condition as “clinically severe” likewise parents of patients who received a psychiatric diagnosis. Patients with externalizing problems were more frequently referred by the parents themselves, while youth with internalizing problems were more often referred through health professionals. Families with non-traditional structures (adoptive, foster care, mono-parental) were more likely to consult CAMHS directly, while immigrant youth were more often referred by teachers.

**Conclusion:**

Socio-demographic and clinical characteristics can affect pathways to care. To improve early access to care for children and adolescents with ongoing mental disorders, a plan for proper action addressed to teachers and health professionals may well be important. This would improve their ability to recognize emotional and behavioral problems and use proper referral pathways, while informative intervention addressed to non-Italian families should inform them about the functioning and the mission of CAMHS.

## Background

Over the past decade, the use of Child and Adolescent Mental Health Services (CAMHS) has increased in many European countries [1, 2], generating requests for additional resources. Nevertheless, a lot of effort is still needed to reduce the “*treatment gap*”. Indeed, only a small portion of children and adolescents with emotional and behavioral problems end up consulting a CAMHS, with percentages ranging from 14–16 % [2, 3], to 20–25 % [4]. Within this general framework, collecting data on *who* and *how* makes contact with CAMHS could contribute to developing effective health service policies and better resource allocation [5].

Problem recognition by parents and service use depends on the parents’ level of education and the amount of stress they experience [6, 7]. It has also been shown that the intensity of treatment is associated not only with the level of psychopathology, but also with the family’s profile [8, 9]. The organization of the healthcare system, specifically the interface between primary and specialist care, has to be considered when dealing with the help-seeking process.

In this regard, the literature has two major limitations: (*i*) most of the published literature is from the US, where there is no National Health Service (NHS) and as a consequence referral sources and pathways to care are different from countries equipped with a public NHS [10]; (*ii*) many studies have been conducted on general population samples, while only a few studies have focused on clinical samples [8, 9].

To overcome these limitations, this study aims to investigate variables related to the process of seeking help by focusing on a clinical sample of Italian children and adolescents at their first contact with CAMHS. Since the access to mental health services in Italy does not necessarily require a referral by GPs or other professionals, and initial assessment (as well as subsequent treatment) is totally free, the proportion of patients referred from other sources was expected to be greater than previously found in other countries with different healthcare systems [11]. Given the ability of healthcare providers to detect psychopathological symptoms more efficiently, we expected that referrals by health professionals would show a higher proportion of clinical diagnoses compared to others.

Moreover, we were interested in comparing the functioning and symptoms of patients with and without a clinical diagnosis when first treated. Given that parents, teachers and health professionals recognize symptoms to varying ability [6, 7], we expected to find an association between referral source and psychopathological symptoms or the reason for consultation. As already mentioned, externalizing symptoms have more chance of being recognized, since they cause disturbances and concern for parents. For this reason we expected that patients with this behavioural pattern would be more likely to be referred by their own family.

Finally, since socio-economic status plays a crucial role in the help-seeking process [6, 7], we expected that higher parental educational level would be associated with more frequent referral by the family. This was because a higher parental educational level could well be related to a better understanding problematic behaviour or symptoms. as well as better knowledge of the health services. For the same reason, we expected that any mental disorders present in parents would result in a higher proportion of referrals by the family. This again was because those who had been diagnosed with a mental disorder may already have some knowledge of the mental health care system.

## Materials and methods

### Setting

The study involved all CAMHSs (*N* = 12; 100 %) of a northern Italian Region, which has approximately 4 million inhabitants; among them there are 633,725 children and adolescents aged 0–17 years. In Italy, CAMHS serve all patients aged between 0 and 18 years suffering from mental or neurological disorders. Access does not require official referrals by GPs. CAMHSs are part of the NHS, and their costs are fully covered by the NHS. A detailed description of the clinical interventions delivered by CAMHS, which participated in the study have been reported elsewhere [12].

### Sample and procedures

In a 6-month period, the first three consecutive patients scheduled for the morning consultation at all participating CAMHSs, were invited to participate in the study. Given the time needed for a thorough evaluation of each patient and his/her family, only the first three consecutive patients could be assessed by the research assistant each morning. In all recruiting centres, a secretary scheduled the appointments only after the request order (generally made by telephone). For this reason, the first three morning visits were a random sample of all visits scheduled each day.

Our Ethics Committees did not allow any information about those patients and their parents who did not agree to be assessed to be recorded or included in the survey. However, these patients were limited ranging from 6 to 11 % depending on the centre. Given the lack of any intervention, the observational nature of the study and the fact that the survey was proposed by the treating clinician, the refusal rate was very low.

Patients were enrolled if the following inclusion criteria were satisfied: age between 6 and 17 years and parents with a good knowledge of written and spoken Italian. Exclusion criteria were: presence of Moderate to Profound Mental Retardation (F71-F73) or Diseases of the Nervous System (G00-G99) as a primary clinical condition. Patients diagnosed with (or suspected of) ADHD and related disorders by the child/adolescence psychiatrist were also excluded from this sample, because in Italy there is a large national project surveying all ADHD patients in treatment. Their inclusion would have replicated an ongoing, larger project on this topic. Interestingly, in the CAMHSs where this study was made, data from the regional registry showed that neurological disorders, mental retardation (F70-79), and ADHD (F90) represent respectively 6.2 % (SD = 5.7)%, 6.6 % (SD = 3.4), 5.8 % (SD = 2.3) of the whole sample who, during 2008, contacted CAMHSs for the first time.

Following these criteria, 699 patients were enrolled. For this paper, only patients at their first-ever consultation with CAMHSs were considered (*N* = 399). The 12 enrolled centers’ institutional review board approved the study protocol, which conformed to the Declaration of Helsinki 1995 (as revised in Tokyo 2004). Parents gave written informed consent. According to our low, we obtained 12 approvals from the 12 centers participating to the study. Every one has a specific name, thus the text may become long.

### Measurements

The ‘Parent Schedule’ is an ad hoc-devised form filled by parents consisting of 59 items covering the following 5 areas: family characteristics, pregnancy and birth, development milestones during early childhood, stressful life-events during the previous year and information about the help-seeking process. As for the latter section, items were formulated to ask about the pathway leading to contact with CAMHS. Parents also reported the reason for consultation, referral source, and previous treatment. The reason for contact was referred by parents by choosing one of the following possible options: low performance at school, refusal to go to school, social withdrawal, physical complaints, low mood, bizarre behaviour, disruptive behaviour and/or language problems. Referral source was rated by choosing from the following options; school, GP or paediatrician, child psychiatrist in private practice, clinical psychologist in private practice, emergency service, nobody.

In addition, parents completed the *Child Behavior Checklist/6–18 (CBCL/6–18)* [13]. The CBCL consists of 113 items grouped into two parts: the first part explores the social competences of children and adolescents, while the second part investigates their behavioural and emotional problems. Many validity and reliability studies have shown that the CBCL is an effective instrument for assessing emotional and behavioural problems in children [13].

The ‘Clinician Schedule’ is an ad hoc-devised form filled by the child psychiatrist who examined the patient. It consists of 41 items covering the following 3 areas: diagnosis, currently planned and previous treatment (both pharmacological and non-pharmacological) and presence of any mental disorders or physical illnesses in parents/siblings. Clinical diagnoses were formulated by the child psychiatrist according to ICD-10 [14].

Concerning familiarity with mental disorders, child psychiatrists rated whether parents or siblings were affected by a mental disorder when a diagnosis had ever been formulated and/or the person had ever been treated. We grouped disorders into the following categories; Mental and behavioural disorders due to psychoactive substance use (F10-19), Schizophrenia, schizotypal and delusional disorders (F20-29), Mood disorder (F30-39), Neurotic, stress-related and somatoform disorders (F40-48), Disorders of adult personality and behaviour (F60-69), Mental Retardation (F70-79). For siblings, we also considered the following disorders; Disorders of psychological development (F80-89) and behavioural and emotional disorders with onset usually occurring in childhood and adolescence (F90-98).

In addition, clinicians rated the following standardized assessment instruments:

- Health of the Nation Outcome Scale for Children and Adolescent mental health (HoNOSCA) [15], consisting of 15 scales, each rated from 0 (no problem) to 4 (severe problem). For this study we used scales 1 through 13 that focused on clinically significant issues and symptoms over the previous 2 weeks [16];
- the Clinical Global Impression Severity Index (CGI-S) [17], a 7-point scale requiring clinicians to rate the severity of a patient’s illness, relative to the clinician’s past experience with patients having similar diagnosis. Scores range from 1 (normal, not ill at all) to 7 (extremely ill);
- Children Global Assessment Scale (C-GAS) [18] is a scale ranging from 0 to 100 to rate general functioning, with higher scores indicating better functioning [16].

### Statistical analyses

All data were coded and analyzed using the Statistical Package for Social Sciences (SPSS) for Windows (Chicago, Illinois 60606, USA), version 13. All tests were two-tailed, with statistical significance set at *p* = 0.05. Categorical data were analyzed into inter-group or intra group comparisons using V Cramer association measures. Multinomial logistic regression, with backward stepwise elimination, was used with “source of referral” as dependent variable. This was categorized as follows; referred by parents themselves, referred by school teachers or referred by health professionals (i.e., they were referred by any of the following professionals: GPs, paediatricians, child psychiatrists in private setting, clinical psychologists in private setting, emergency services).

The following variables were entered; age, symptom severity, reason of contact, family structure, parents’ educational level and presence of mental disorder in parents/siblings. Age ranges from 6 to 18 years old and we categorized them into four classes (6–8, 9–10, 11–13, 14–18) to distinguish between children, pre-adolescents, and adolescents. Symptom severity was described by CBCL internalizing, externalizing and total score, which respectively represent the intensity of internalizing, externalizing and general symptoms severity. According to the scoring system, each of the three scores can be categorized into normal, borderline, or clinical areas.

For our analysis, we compared clinical scores versus borderline or normal scores. Reasons for contact were referred by parents by choosing one of the possible options; low mood (including low mood, social withdrawal and physical complaints), school problems (including refusal to go to school, low performance at school) and behavioural problems (including bizarre behaviour, rule-breaking behaviour, and language problems). Concerning family structure, according to Sourander et al. [1] and Tick et al. [2], we compared traditional (i.e., family biological parents) versus non-traditional families (i.e., one-parent families, one-step parents, adoptive parents, foster family, child/adolescent living in a residential facility). Parents’ educational level was divided into bachelor versus less than bachelor’s degree. As for the familiarity for mental disorder, the presence or absence of any mental disorders was entered.

Area Under the Curve (AUC) values were reported in order to quantify the performance of standardized assessment instruments (CBCL Total Problem, CBCL Performance Score, C-GAS, CGI, HoNOSCA). AUC is a non-parametric value and indicates the performance of a binary classifier system. The reported effect size intervals for AUC were: small effect sizes (0.52–0.55), medium effect sizes (0.56–0.69), large effect sizes, (0.70–0.76), very large effect sizes (above 0.76) [19].

## Results

### The sample

The mean (SD) age of the sample was 10.5 (3.2) years. Most participants were living with their biological family (*N* = 302, 75.7 %) (Table 1). Those adopted or living in a foster care family (*N* = 34, 8.5 %) started living with their new family on average at 3 years of age (SD = 2.1). Patients of non-Italian nationality (*N* = 27; 6.8 %), on average had been living in Italy for 6 years (SD = 4). Stressful life events during the past year before the consultation were reported by one third of families: in 132 (33.2 %) cases parents reported economic difficulties (e.g., substantial reduction of family income and/or debts), in 112 (28.1 %) cases parents reported school difficulties (e.g., starting a new school, problems with the teacher) as the main stressful life event and for 110 (27.2 %) children/adolescents, parents mentioned relational or emotional problems in the family (e.g., separation, bereavement).

**Table 1:** Patients at first-ever consultation with child and adolescent mental health services

		*N* (%)
Sex	Male	227 (56.9)
Age (years)	6–8	138 (34.6)
	9–10	74 (18.5)
	11–13	106 (26.6)
	14–18	85 (20.3)
Nationality	Italian	372 (93.2)
	European Union (EU)	15 (3.8)
	Non-EU	12 (3.0)
Family structure	Traditional structure (father, mother, siblings)	307 (75.7)
	Non-traditional structure:	
	One-parent family	58 (14.5)
	One step-parent	26 (6.5)
	Adoptive or Foster family/Residential care	8 (2.0)
Diagnosis	F40-48 Neurotic, stress-related, somatoform disorders	68 (17.0)
	F93-98 Emotional disorders with childhood onset	67 (16.8)
	F30-33 Mood disorders	19 (4.8)
	F81 Learning disorders	84 (21.1)
	F80 Specific disorders of speech and language	19 (4.8)
	F82, 83, 88 89 Other developmental disorders	6 (1.5)
	F70 Mental retardation	17 (4.3)
	F84 Pervasive developmental disorders	9 (2.3)
	F91-92 Conduct disorders	25 (6.3)
	F50 Eating Disorders	24 (6.0)
	F60-69 Disorders of adult personality and behaviour	5 (1.3)
	F20-29 Schizophrenia, schizotypical and delusional	5 (1.3)
	Z03-99 Factors influencing health and contact with health services	51 (12.7)
Familiarity for mental disorders and physical illnesses	Mental disorder in one parent	89 (22.3)
	Mental disorders in both parents	24 (6.0)
	Physical illness in one parent	60 (15.0)
	Physical illness in both parents	12 (3.0)
	Mental disorders in any brothers/sisters	44 (11.0)
Main scores on assessment questionnaires		Mean (SD)
Child Behavior Checklist/6–18 Total performance score		16.4 (6.0)
Child Behavior Checklist/6–18 Total problem score		47.9 (24.6)
Clinical Global Impression Severity Index		3.1 (1.2)
Children Global Assessment Score		64.2 (14.1)
Health of the Nation Outcome Scale for Children and Adolescent		8.6 (5.6)

### Source of referral to CAMHS and reason of contact

Most parents reported they had been referred to CAMHS by their child’s school teacher (*N* = 145; 36.4 %) and approximately 17 % of parents (*N* = 70) had directly requested a CAMHS consultation with no referral from other health professionals or teachers (Table 2).

**Table 2:** Access to child and adolescent mental health services

		*N* (%)
Reasons for consultation	Low performance at school	199 (50.4)
	Low mood	67 (17.0)
	Social withdrawal	45 (11.4)
	Language problems	42 (10.6)
	Bizarre behaviour	42 (10.6)
	Refusal to go to school	38 (9.6)
	Physical complaints	34 (8.6)
	Rule-related problem	19 (4.8)
Source of referral	School	145 (36.4)
	General Practitioner or Paediatrician	130 (32.7)
	Parent themselves	70 (17.6)
	Child psychiatrist or clinical psychologist in private practice	41 (10.3)
	Emergency service	20 (5.0)
Treatment prior to CAMHS referral	No previous treatment	277 (69.4)
	Treatment by Child psychiatrist or clinical psychologist in private practice	57 (17.1)
	Treatment by General Practitioner or Paediatrician	53 (16.8)
	Treatment by Family counselling service	10 (3.0)

Half of the patients were referred to CAMHS for poor school performance (*N* = 199; 50.4 %). A total of 277 (69.4 %) patients had received no previous treatment before their first CAMHS contact, while 57 (17.1 %) patients had been treated by private child specialists before CAMHS consultation.

### Patients contacting CAMHS without an initial diagnosis of mental disorder

The proportion of first-ever contacts who did not receive any diagnosis of mental disorder at their first consultation did not differ by source of referral (V Cramer = 0.025, *p* > 0.05) (Table 3).

**Table 3:** Comparison of scores of assessment instruments between youths with and without a clinical diagnosis at first evaluation

Assessment instrument	Subjects without a clinical diagnosis (mean + SD)	Subject with a clinical diagnosis (mean + SD)	AUC value^a^ (*p*)
CBCL Total Problem	39.9 (21.8)	45.3 (29.9)	AUC = 0.54 (*p* = 0.404)
CBCL Performance Score	17.6 (5.5)	16.1 (6.0)	AUC = 0.42 (*p* = 0.125)
Children Global Assessment Scale	74.1 (11.6)	63.0 (13.9)	AUC = 0.72 (*p* < 0.001)
Clinical Global Impression Severity Index	2.1 (0.8)	3.2 (1.2)	AUC = 0.78 (*p* < 0.001)
Health of the Nation Outcome Scale for Children & Adolescent	4.9 (3.9)	9.0 (5.5)	AUC = 0.74 (*p* < 0.001)

^a^AUC values are reported in order to quantify the performance of standardised assessment instruments (CBCL Total Problem; CBCL Performance Score; C-GAS; CGI; HoNOSCA). AUC is a non-parametric value and illustrates the performance of a binary classifier system; reported effect size intervals for AUC are: small effect sizes (0.528–0.556); medium effect sizes (0.584–0.638); large effect sizes. (0.714–0.760); very large effect sizes (above 0.760)

Those participants who received a clinical diagnosis were compared to those who did not satisfy criteria for any mental disorder according to the child psychiatrist’s initial clinical diagnosis. These two groups were compared on the scores of assessment instruments covering clinical symptoms and psychosocial functioning. There were significant differences on clinician-rated instruments (e.g., C-GAS, CGI, and HoNOSCA) indicating worse functioning in those with clinician-diagnosed mental disorders (AUC > 0.7; large effect size), while no statistically significant differences were found on parents-rated CBCL scores.

### Socio-demographic and clinical variables associated to source of referral

The source of referral turned out to be significantly associated with specific predictive factors, including likelihood ratio test (*χ*^2^ = 57.32; *p* < 0.0001). In more detail, children and adolescents referred by parents themselves were twice more likely to exhibit clinical scores on CBCL externalizing problems (OR = 2.76; CI 1.21–6.30; *p* = 0.02) compared to those referred by health professionals (Table 4). Patients referred by school teachers were three times more likely to be non-Italian (OR = 3.70; CI 1.08–12.65; *p* = 0.04) compared to those referred by health professionals. Moreover, school teachers were statistically less likely than health professionals to refer for assessment and care any children or adolescents with clinical scores on CBCL internalizing problem (OR = 0.34; CI = 0.17–0.67; *p* < 0.001) or to require a consultation for mood-related problems (OR = 0.24; CI = 0.09–0.61; *p* < 0.001).

**Table 4:** Variables associated to the source of referral

Dependent variable	Independent variables	*p*	O.R.	95 % CI
Referred by parents themselves	Borderline externalizing CBCL vs normal score	0.016*	2.7	1.2–6.3
	Clinical externalizing CBCL vs normal score	0.096	2.1	0.8–4.8
	Non-traditional family vs traditional	0.06	7.1	0.95–45.4
	Consultation for low mood vs any other reason	0.623	0.8	0.38–1.7
	Non-Italian nationality vs Italian	0.169	0.2	0.01–2.1
	Borderline internalizing CBCL vs normal	0.693	0.8	0.4–1.84
	Clinical internalizing CBCL vs normal	0.786	0.86	0.30–2.4
Referred by school teachers	Borderline externalizing CBCL vs normal	0.222	1.6	0.75–3.4
	Clinical externalizing CBCL vs normal	0.288	0.96	0.23–1.5
	Non-traditional family vs traditional	0.409	0.41	0.05–3.3
	Consultation for low mood vs any other reason	0.003*	0.24	0.09–0.61
	Non-Italian nationality vs Italian	0.037*	3.7	1.0–12.6
	Borderline internalizing CBCL vs normal	0.629	0.82	0.37–1.80
	Clinical internalizing CBCL vs normal	0.002*	0.34	0.17–0.66

Multinomial logistic regression, with backward stepwise elimination, was used to assess the association between the dependent variable “source of referral” (*referred by parents* and *referred by school teachers*; reference category: referral by professionals), and the following predictive factors: CBCL internalizing, externalizing and total scores, reasons of contact, family structure, and nationality

* *p* ≤ 0.05

## Discussion

This study investigated the pathways to care to CAMHS and the source of referral. It also studied their association with reasons for contact, as well as with sociodemographic and clinical characteristics, in a sample of children and adolescents at their first-time contact with CAMHS. Moreover, we compared psychosocial functioning and symptoms of those with and without initial clinical diagnosis for mental disorders, in order to understand the needs of families who request a consultation with CAMHS.

### Source of referral

Most patients had been referred by health care professionals and, as we expected, the proportion of patients referred by parents (about 17 %) or referred by schools (about 36 %) was greater when compared to the percentage reported for countries where GPs have a greater gate-keeping role. In a British study, the authors found that only 1.4 % of patients were self-referred and an additional 7 % were referred by educational services [11]. In our country, teachers’ perception of problem issues, their ability to recognize psychopathological symptoms and the advice they can offer to parents are probably influential in guiding children and adolescents to mental health care. Moreover, as parents appear to follow teachers’ advice, proper action is often addressed to teachers in order to improve their ability to recognize emotional signs and behavioural problems about mood disorders.

A substantial proportion of our sample (30 %) received treatment by a GP or by a private child specialist before consulting CAMHS. It seems unlikely that the first consultation with a private specialist was due to a delay of the first visit in the public healthcare sector, causing a diversion to the private health practice, as most units guarantee an initial visit within 2 weeks from the request [12]. We can only hypothesize that stigma associated with public mental health services could induce parents of children and adolescents with emotional problems initially to seek help elsewhere (i.e., private psychiatrists or psychologists, school counselors, etc.) before consulting CAMHS. It is also possible that specific supportive interventions (such as school support, domiciliary care, etc.) are available only from public services and this may motivate request for consultations with CAMHS after a first consultation with a private practitioner. Future qualitative studies are needed to understand parents’ opinion about barriers to care and how to facilitate early contact and care with CAMHS.

### Patients contacting CAMHS without an initial diagnosis of mental disorder

Given that in the Italian mental health system there is no filter to specialist care, we believed that the proportion of patients not satisfying clinically-based criteria for any mental disorders at the first-time consultation would be lowest among those referred by health professionals. Contrary to our expectations, we did not find any significant difference in this clinical subgroup by source of referral. Interestingly, parents of children who did not receive a clinical diagnosis of mental disorders described them as “psychosocially impaired” and their condition as “clinically severe”, likewise the parents of those who received a psychiatric diagnosis, while with clinicians reported different levels of clinical severity for the two groups of patients.

This result highlights the importance of an assessment based on multiple informants and multiple information sources, not limited to the clinician-based diagnosis. This has relevant clinical implications particularly for those countries (like Italy), where clinicians are not used to employ standardized assessment instruments during the evaluation process. Moreover, based on parent-rated assessment instruments, we can argue that “need for care” cannot be limited to psychiatric diagnoses. The simple presence of a mental disorder, clinically evaluated, may not be a sufficient criterion to determine the appropriateness of a referral and consequently appropriate resource allocation.

In the same way, in adult psychiatric populations, it has been demonstrated that illness severity is not simply a matter of diagnosis, but is related to a variety of psychosocial variables [20]. Previous studies on young clinical populations have also found that beyond psychopathology itself, socio-demographic variables [21] and family characteristics [9] influence the level of treatment received by children and adolescents with emotional and behavioural problems.

### Clinical variables associated with the source of referral

Our results show that the contact pattern with CAMHS differs, depending on the presence of externalizing or internalizing problems. We found that patients with externalizing problems were more frequently referred by parents themselves, while youths with internalizing problems were more often referred by health professionals. Finally, teachers generally suggested consultation of mental health services mainly for educational issues. Previous studies have investigated the role played by the type and severity of psychopathology in the help-seeking process. Available evidence supports the hypothesis that there is a better chance of problem recognition in the case of externalizing problems [6, 7]. However, there are studies which found different results, highlighting the role of other variables such as socioeconomic status [20] and the point of view of persons involved in the assessment of child behaviour [21].

Essentially, teachers, parents and health professionals differ in their ability to recognize children’s issues and needs. This underlines the need to educate parents and train teachers regarding timely identification of mental disorders, above all of emotional issues, since the detection of an internalizing problem is more difficult than in the case of an externalizing problem [7].

### Sociodemographic variables associated with source of referral

We found that children of foreign families were more frequently referred to CAMHS by teachers. Although this association is to be treated with caution in light of the low proportion of immigrant family in our sample, we can suggest that they face more prominent educational issues because of language-related difficulties or social inclusion issues. This finding may also indicate some difficulties in problem recognition by parents, or greater negative social stigma for their children when accessing mental health care. Whatever the reason, school plays a crucial role in detecting and referral of young persons with emotional or behavioral issues belonging to minority ethnic groups, since their parents may have limited information about the function of mental health services. As a result informative intervention by public health authorities specifically addressed to immigrant groups to inform them about CAMHS could be important.

In our sample, families with a non-traditional structure (e.g., one-parent, one-step, adoptive or foster family), seem to contact services on their own initiative, without any mediation. A possible explanation of this phenomenon may be their higher acquaintance with specialists and welfare services, possibly due to past events related to the adoption process or divorce, during which they came into contact with these services and gained trust in them. Single and adoptive parents may also suffer greater stress from their children’s behavioral problems and might be more sensitive to their children’s difficulties.

Our results seems to be in line with previous studies that point to the fact that families with a single-parent or a family structure different from a two-biological parent family appear to be more inclined to get in contact with CAMHS, even controlling for children’s symptom severity [1, 2]. Similarly, it has been found that adopted and foster care family children are more likely to have contact with mental health services than biological family children [22] and adopted children more frequently have problems compared to their biological family peers [23].

### Limitations

There are limitations of this study which have to be considered. First of all, patients with neurological disorder, ADHD and Mental Retardation were excluded, which constitutes a limit for a study focused on patterns of access to CAMHS. However we reported registry data about the overall annual prevalence of children/adolescent with these disorders. In addition, subjects with ADHD are presently included in an ongoing national epidemiological study. Moreover, we evaluated children and adolescents in the age range 6–17 years. As a result, children with severe developmental disorders, such as autism or intellectual disabilities, were most probably excluded because they have already accessed the system at an earlier age.

We also excluded those who did not have good knowledge of written and spoken Italian, limiting the generalizability of our results to non-Italian subjects. The diagnoses were based on clinical assessment, and can be considered the main limitation of this study. Indeed, only a moderate association has been found between clinical diagnoses and standardized diagnostic interviews [24]. However, in the busy daily setting of the CAMHS, where the study was carried out, it was impossible to administer structured interviews to a large group of patients, such as those evaluated in this survey.

Finally, it would have been interesting to test an association between stressful life events and previous treatment with the source of referral. Unfortunately we had no data to explore such an association. As for fee for treatment, the initial evaluation is free so we can exclude that this variable had any influence on the initial intake.

## Conclusion

Although teachers do not play a gatekeeper function, our study highlights their important role in the help-seeking process. This would suggest the importance of proper action addressed to teachers in order to improve their ability to recognize emotional and behavioral problems. Moreover, informative intervention addressed to foreign family may be helpful to let them know the functioning and mission of CAMHS. Finally, future studies should analyze parents’ opinion on barriers to care and how to facilitate early contact and care with CAMHS.
